# Sciatic nerve block or not for outpatient total knee arthroplasty? Study protocol for a randomized controlled trial

**DOI:** 10.1186/s13063-018-3142-1

**Published:** 2019-01-08

**Authors:** Laurie Tran, Melissa Barthelemy, Pascal Boileau, Marc Raucoules-Aime, Michel Carles, Christophe Trojani

**Affiliations:** 1 Department of Anesthesiology, Intensive care and Emergency Medicine, Pasteur 2 Hospital, 30, Voie Romaine, CS 51069, 06001 Nice, Cedex 1 France; 2 Department of Orthopedics & Sports, iULS - University Institute of Locomotion & Sports, Pasteur 2 Hospital, 30, Voie Romaine, CS 51069, 06001 Nice, Cedex 1 France

**Keywords:** Total knee arthroplasty, Outpatient procedure, Sciatic nerve block, Randomized controlled study

## Abstract

**Background:**

The number of patients operated on for total knee arthroplasty (TKA) is growing worldwide. Outpatient surgery is defined by a length of stay (LOS) in the hospital of less than 12 h. This can be limited for TKA, with the efficient management of pain and perioperative complications, such as blood loss, affecting a safe hospital discharge. Outpatient TKA with a suitable protocol, including multimodal measures, could improve the success rate of this procedure. Among the main measures, single-shot sciatic nerve block in association with continuous femoral nerve block for pain control needs to be evaluated in outpatient TKA. Furthermore, to promote the safety of the postoperative period and to accelerate rehabilitation, patients who undergo ambulatory TKA could be discharged to a rehabilitation center on the day of surgery to screen adverse events and to optimize the rehabilitation process. This study is designed to assess the benefits of sciatic nerve block in postoperative pain relief for outpatient TKA.

**Methods/design:**

This randomized prospective controlled study will be conducted in the knee unit of the teaching hospital of the Nice university and will include 40 patients undergoing primary unilateral outpatient TKA, discharged the day of surgery to a private rehabilitation center for enhanced recovery after surgery, after a hospital stay of less than 12 h. Before surgery, all patients will receive a continuous femoral nerve block with 2 mg/ml ropivacaine 20 ml, and then patients will be randomly assigned to receive or not receive a single-shot sciatic nerve block with 2 mg/ml ropivacaine, 20 ml. The primary outcome measure is the success rate of outpatient TKA. This rate is defined by patients discharged from the hospital to a rehabilitation center the day of surgery with no re-hospitalization due to insufficient pain control before the fifth postoperative day. Secondary outcomes include the incidence of major and minor adverse events during the first five postoperative days and measurement of the quality of recovery using the Knee injury and Osteoarthritis Outcome Score and the new International Knee Society scores plus the Quality of Recovery-40 questionnaire.

**Discussion:**

The assessment of anesthesia and rehabilitation protocols enabling major orthopedic surgery, such as TKA, is necessary. This randomized controlled study will address the hypothesis that a suitable multimodal protocol including sciatic nerve block could improve pain control and thus improve the success rate of outpatient TKA.

**Trial registration:**

EudraCT, 2016-000226-19. Registered on 15 April 2016.

**Electronic supplementary material:**

The online version of this article (10.1186/s13063-018-3142-1) contains supplementary material, which is available to authorized users.

## Background

Total knee arthroplasty (TKA) is commonly performed to relieve pain due to osteoarthritis, to improve knee function, and to increase quality of life [1]. The concept of fast-track surgery described by Henrik Kehlet in 2008 is known to reduce perioperative mortality and morbidity [2, 3]. Since the last decade, fast-track surgery with multimodal perioperative care has been applied to this major orthopedic procedure to reach an ultimate goal: outpatient TKA surgery [4–6].

In outpatient TKA, patient selection is also strongly recommended [4–6], because preexisting comorbidities and advanced age are associated with a higher prevalence of postoperative complications.

Outpatient surgery is defined by a length of stay (LOS) in the hospital of less than 12 h [7], but it can be limited for TKA by adequate management of pain [8, 9] and other perioperative complications, such as blood loss [10], affecting a safe hospital discharge. For pain control, the efficacy of continuous femoral nerve block in TKA is well documented; it enables a controlled opioid consumption [11–13] and could reduce hospital LOS [14]. The benefits of sciatic nerve block in association with continuous femoral nerve block has been proven recently [15–17], but not in outpatient surgery. Postoperative anemia also limits outpatient procedures [18, 19]. Therefore, a strategy limiting perioperative blood loss including biological and clinical monitoring is mandatory.

In the published studies on TKA, almost 90% of patients identified as “outpatients” spent 23 h in the hospital prior to discharge to their homes [20–22] and could therefore be considered as “inpatients”, as they stayed one night in the hospital. In the present study, to obtain a hospital LOS shorter than 12 h, patients will be discharged directly to a rehabilitation center. This strategy ensures complete screening of postoperative complications and allows an enhanced recovery after surgery (ERAS).

Major adverse events or readmissions are not different in outpatient procedures compared to inpatient procedures [21]. Finally, outpatient TKA surgery reduces health care costs [22–24].

This study is designed to assess the benefits of sciatic nerve block in postoperative pain relief for outpatient TKA. We also investigate the incidence of adverse events such as hematoma, anemia, thromboembolism, and surgical site infection in the first 5 postoperative days.

## Trial design and methods

### Study design

**Fig. 1:**
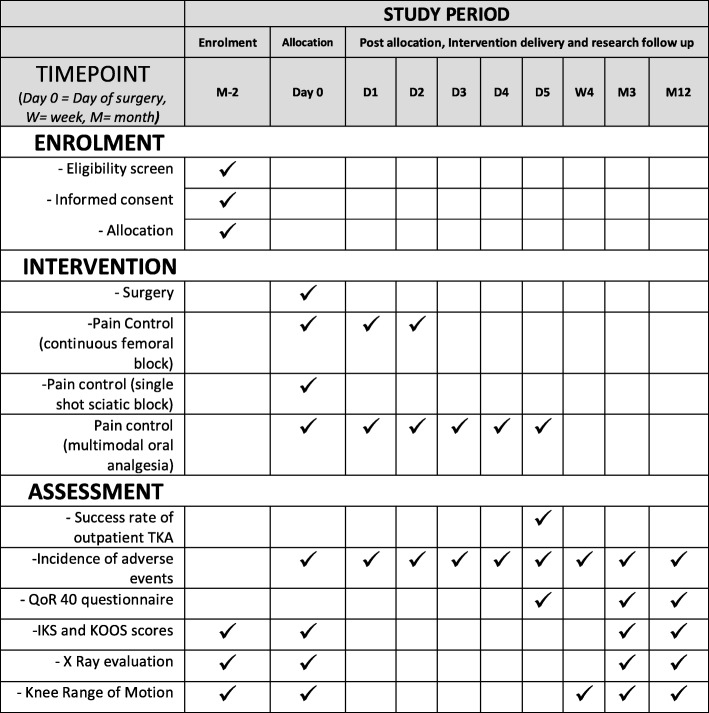
Outpatient TKA procedure SPIRIT figure: schematic diagram of enrollment, interventions, and assessments of trial participants in outpatient TKA protocol

This single-center randomized prospective controlled trial respects the Declaration of Helsinki and is approved by the ethics committee “Sud Mediterranee V” and the French National Drug Security Agency (160095A-31). Written informed consent will be obtained from each patient before enrollment. This study follows the Consolidated Standards of Reporting Trials (CONSORT) and the Standard Protocol Items: Recommendations for Interventional Trials (SPIRIT) 2013 Statements, Fig. 1 (the SPIRIT checklist is available as Additional file 1) [25, 26]. The trial is registered in EUDRACT, number 2016-000226-19 (registered on 15 April 2016).

**Fig. 2:**
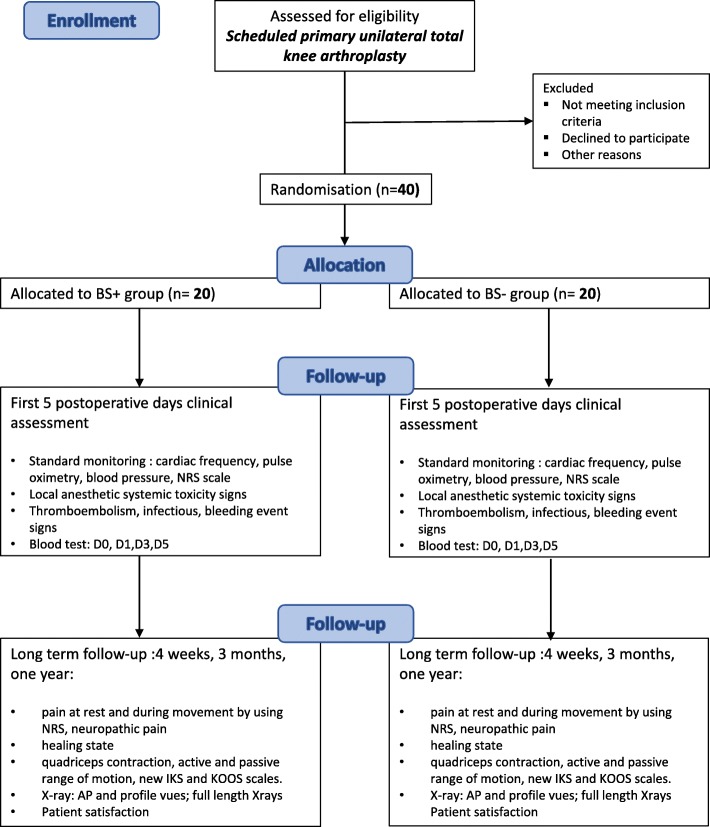
Overview of study protocol. *BS+ group* group with sciatic nerve block (single shot) and continuous femoral nerve block, *BS– group* group with only continuous femoral nerve block

An overview of the trial design is shown in Fig. 2.

### Recruitment and consent

We will recruit 40 patients through the University Institute of Locomotion & Sports of Pasteur 2 Hospital in Nice, France. Consecutive patients presenting for primary unilateral TKA will be screened for eligibility. Patients not participating in the study will receive usual care (TKA inpatient).

Those willing to participate will be asked for informed consent using an approved consent form.

To be eligible, participants must meet the following inclusion criteria:

- Scheduled primary unilateral TKA
- Acceptance of outpatient procedure
- Patient age above 18 years and under 75 years
- Normal weight or moderate obesity
- American Society of Anesthesiologists (ASA) classification 1–2
- Patients without cognitive disorders having a good level of understanding of outpatient procedure (pain control)
- No major thromboembolic episode in medical history
- No contraindications for anesthesia or analgesia
- Preoperative hemoglobin > 13 g/dl

Exclusion criteria will include:

- Bilateral TKA
- Revision TKA
- Unicompartmental knee arthroplasty
- Severe (body mass index (BMI) > 35) and morbid (BMI > 40) obesity
- ASA classification 3–4
- Obstructive sleep apnea syndrome
- Hypersensitivity or allergy to anesthesia drugs
- Refusal of the outpatient procedure by the patient
- Emergency surgery
- Anticoagulant therapy

### Preoperative surgical evaluation

To assess pain and disability associated with knee osteoarthritis, the Numeric Rating Scale (NRS), the new Knee Society Scoring (KSS) System [27], and the Knee injury and Osteoarthritis Outcome Score (KOOS) [28] will be systematically completed for each patient during the preoperative time. Furthermore, X-ray evaluation will include the anterior to posterior (AP) view and lateral and patellar views for each patient, as well as full-length X-rays to evaluate varus and valgus deformities. 

### Randomization and group allocation

The objective of this study is to assess a protocol for outpatient TKA including postoperative analgesia optimized to allow same-day discharge to a rehabilitation center. For TKA, all patients usually receive continuous femoral nerve block with 2 mg/ml ropivacaine. Two groups are defined randomly. Patients who receive sciatic nerve block with 20 ml of ropivacaine 2 mg/ml are allocated to the “sciatic block group” (BS+). Patients who will not receive sciatic nerve block are allocated to a second group (BS–). Random assignment is performed before surgery using a computer-generated randomization table.

### Anesthesia and analgesia

**Fig. 3:**
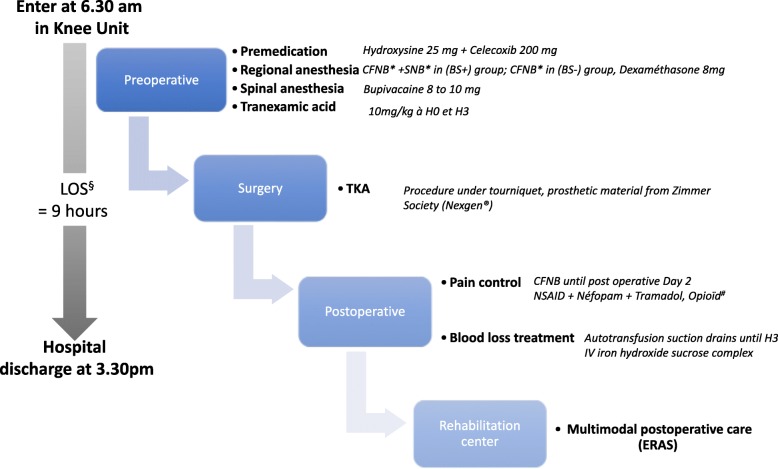
The “outpatient TKA journey”; ^*^CFNB (continuous femoral nerve block) and *SNB* (sciatic nerve block) performed with ropivacaine 2 mg/ml, 20 ml, ^§^*LOS* hospital length of stay, ^#^*Opioïd* oral morphine sulfate, if NRS > 30

The “outpatient TKA journey” is represented in Fig. 3. Premedication by oral dose of celecoxib 200 mg [29] and hydroxysine 25 mg will be administered in the surgical care unit. All recruited patients will receive continuous femoral nerve block (under ultrasound guidance [30]) with 2 mg/ml ropivacaine, 20 ml. In the BS+ group, patients will also receive a sciatic nerve block (subgluteal approach [31] under ultrasound guidance) with 2 mg/ml ropivacaine 20 ml. In order to improve quality and duration of the nerve block, intravenously administered (IV) dexamethasone 8 mg will be administered at the time of regional analgesia [32]. Surgery will be performed under spinal anesthesia (L3–L4 level, in lateral decubitus, on the same side as the surgery) with 8 or 10 mg of bupivacaine 5 mg/ml respectively, for women and men.

For all patients, prophylactic antibiotic treatment with IV cefazolin 2 g will be given 30 min before surgery. The prophylactic antibiotic treatment will be continued with oral clindamycin (600 mg every 8 h) until postoperative day 1, as suggested by the French Society of Anesthesiology.

The first IV analgesic will be administered after skin incision with paracetamol 1 g and nefopam 20 mg.

### Surgical procedure and blood loss treatment strategy

All patients will receive two infusions of tranexamic acid 10 mg/kg (before surgery and at the third postoperative hour). A Zimmer NexGen® prosthesis will be implanted in all recruited patients. The procedure is performed using a 250 mmHg tourniquet, from skin incision to knee compression dressing, through a medial approach in a varus knee and a lateral approach in a valgus knee. The NexGen prosthesis is postero-stabilized with a fixed polyethylene bearing. The patella will be resurfaced systematically. Femoral, tibial, and patellar components will be cemented systematically with Palacos® gentamicin. Two intra-articular autotransfusion suction drains will be systematically positioned: the suction drains will be removed after the first passive physiotherapy session (Kinetech®) with dressing refection (semicompressive) at the third postoperative hour. Autotransfusion will be infused if the total blood volume is above 300 ml. IV iron (iron hydroxide sucrose complex 300 mg) will be infused in the post-anesthesia care unit (PACU) and on the second postoperative day.

### Postoperative analgesia

Pain at rest and during movement will be assessed by using the NRS.

Multimodal pain management will include:

- Continuous femoral nerve block with infusion of 2 mg/ml ropivacaine by using a perineural patient-controlled analgesia with continuous infusion (5 ml/h) and bolus (5 ml every 30 min). Perineural infusion will run until the second postoperative day.
- Paracetamol 1 g/8 h
- Ketoprofen 100 mg/12 h

A rescue analgesic protocol will be performed if pain control proves to be insufficient (NRS > 30) 20 min after ropivacaine bolus by using IV tramadol 100 mg.

If the NRS value remains > 30 at 20 min after tramadol infusion, oral morphine sulfate 10 mg will be administered to the patient. This analgesic routine will be repeated until NRS < 30.

### Early mobilization

All patients will be seen by a physiotherapist the day of surgery to start the rehabilitation program with passive physiotherapy and ambulation using a walker: at 3 h post-surgery, a physiotherapy session will be performed; then the dressing will be changed and the drains will be removed, and the patients will walk with the help of a physiotherapist.

### Hospital discharge to a rehabilitation center

Patients will be discharged to a rehabilitation center on the same day as surgery if the following points are checked and met:

- Ramsay scale = 2 [33]
- Modified Aldrete scale = 10 [34]
- No adverse events: no postoperative vomiting and nausea, no agitation, no urinary retention, normal blood pressure, normothermia, cardiac frequency < 130% of preoperative level, NRS < 30
- Postoperative hemoglobin level > 11 g/dl
- Standing position and walk with crutches, and Zimmer splint performed by patient
- Patient consent to be discharged from the hospital to the rehabilitation center (written consent also signed by the surgeon and anesthetist performing the procedure)

### Follow-up in rehabilitation center

Patient follow-up will be performed by a multidisciplinary team (surgeon, anesthetist, physical therapist, and nurses).

Standard monitoring from postoperative day 1 to day 5 will be evaluated (cardiac frequency, pulse oximetry, blood pressure, pain with NRS assessment).

Clinical monitoring of the continuous femoral nerve block and the sciatic block according to the French Society of Anesthesiology recommendations will be performed: search for signs of local anesthetic systemic toxicity (central nervous system manifestations with circumoral and/or tongue numbness, metallic taste, lightheadedness, dizziness, visual or auditory disturbances, drowsiness, seizure, unconsciousness; cardiovascular manifestations with collapse).

Clinical signs of a thromboembolic event (pain, tenderness, swelling, discoloration in the lower extremities, dyspnea, tachypnea, chest pain), infectious complications, and bleeding (hematoma) will be assessed as well. A complete blood count (CBC) will be performed on postoperative day 1, day 3, and day 5. Patient satisfaction will be evaluated with the Quality of Recovery (QoR)-40 score [35].

### Long-term follow-up

Patients will return for medical and surgical follow-up at 4 weeks, 3 months, and 1 year to assess knee mobility with quadriceps contraction, active and passive range of motion, pain at rest and during movement measured using NRS, and assessment of residual neuropathic pain (DN4 questionnaire) [36]. The new International Knee Society (IKS) and KOOS scores will be systematically completed during the postoperative period. Furthermore, X-ray evaluation will include an AP view, lateral and patellar views, and a full-length X-ray to help assess residual varus and valgus deformities. Patient satisfaction with the outpatient procedure will be assessed using the QoR-40 questionnaire.

### Primary outcome evaluation

The primary outcome is the success rate of outpatient TKA. The success rate is defined by patients discharged from the hospital to a rehabilitation center the day of surgery with no re-hospitalization due to insufficient pain control until the fifth postoperative day.

### Secondary outcomes evaluation

Secondary outcomes include the incidence of adverse events before the fifth postoperative day (including hematoma, anemia, thromboembolism event, surgical site infection) and measurement of quality of recovery using the QoR-40 questionnaire.

### Sample size

Calculation of the sample size was based on previous studies [12, 37].

We assumed that the failure of the outpatient TKA procedure, defined by no discharge the day of surgery or re-hospitalization during the postoperative period due to pain, could reach a peak value of 55%. The association of sciatic nerve block and continuous femoral nerve block could decrease this failure rate up to 15%. Group sample sizes of 19 for each group will achieve 80% power to detect this difference rate of outpatient procedure failure with an α risk of 5%. We will include 40 patients (20 in each group).

### Data analysis

Data are expressed as mean ± standard deviation (SD), median (interquartile range (IQR)), or percentage. Continuous variables will be analyzed with a two-sample *t* test with equal/unequal variance or with a Mann and Whitney *U* test, if appropriate. A chi-square test or Fischer’s exact test will be used for categorical variables. NRS will be analyzed with repeated measures for general linear models and multivariate analysis of variance. A Bonferroni correction will be applied for multiple comparison and subgroup analyses. *P* values < 0.05 will be considered significant. All statistical analyses will be performed using R Studio®, version 1.

## Discussion

This single-center randomized controlled study will provide clinical evidence on the efficacy of a secure protocol for outpatient TKA: the expected results will help to establish the best inclusion criteria for this procedure. Indeed, to anticipate medical adverse events due to patient comorbidities, strict patient selection is mandatory. Therefore, we decided to only include patients under 75 years of age, of ASA classification 1–2, with no comorbidities. Furthermore, to prevent medical complications resulting from surgery, specifically trained teams and a suitable protocol are recommended. Key elements of these protocols include precise preoperative evaluation, standardized analgesic/anesthetic regimens, standardized surgical procedures, and early mobilization. Finally, discharge from the hospital the day of surgery to a rehabilitation center improves the safety of the postoperative period and allows rapid recovery after surgery. It also inspires confidence in patients and health caregivers, even if the term “outpatient” could be challenged, as patients are not discharged to their homes.

This study aims to assess a LOS of less than 12 h in the hospital after a total knee replacement. If the results show that this protocol is safe, the next step will be to discharge patients with the same inclusion criteria directly to their homes.

### Trial organization

In June 2016 ethical approval was granted by the “Sud Mediterranean V” Regional Research Ethics Committee.

In July 2016 recruitment started.

In September 2016 the first patient was randomized.

In December 2016 the first patient completed the 3 months’ follow-up.

In April 2018 recruitment was completed.

In June 2018 the last patient was randomized.

In September 2018 the last patient completed the 3 months’ follow-up. Analysis and publication of outcome data proceeded.

## Additional file

Additional file 1: SPIRIT 2013 checklist: recommended items to address in a clinical trial protocol and related documents. (DOC 145 kb)
