# Supplementary material for: Sciatic nerve block or not for outpatient total knee arthroplasty? Study protocol for a randomized controlled trial
Source: Trials. 2019 Jan 8;20:30. doi: 10.1186/s13063-018-3142-1 (PMC6325783; doi:10.1186/s13063-018-3142-1)
Supplement: Supplementary file 1 — SPIRIT 2013 checklist: recommended items to address in a clinical trial protocol and related documents. (DOC 145 kb) [file 13063_2018_3142_MOESM1_ESM.doc]

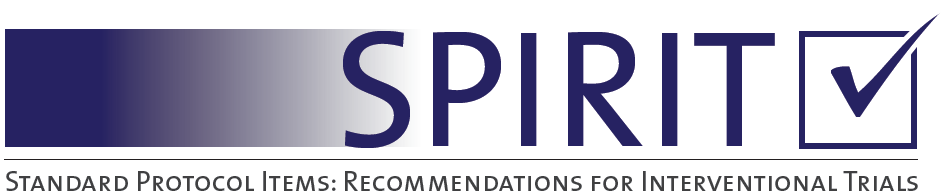


SPIRIT 2013 Checklist: Recommended items to address in a clinical trial protocol and related documents*

| Section/item | ItemNo | Description |
| --- | --- | --- |
| **Administrative information** | | |
| Title | 1 | **Sciatic Nerve Block or not for outpatient Total Knee Arthroplasty? Study protocol for a randomized controlled trial** |
| Trial registration | 2a | Ethics committee of the French “SUD MEDITERRANNEE V”( number 16.018), French National Drug Security Agency (160095A-31)  EUDRA CT, number 2016-000226-19 (April 15^th^ 2016) |
|  | 2b | No WHO registration |
| Protocol version | 3 | Protocol version 3, 07/06/2016 |
| Funding | 4 | No Funding |
| Roles and responsibilities | 5a | **Conception of the study**: Christophe Trojani MD, PhD (iULS - University Institute of Locomotion & Sports, Department of Orthopedics & Sports, General and teaching Hospital of Nice); Melissa Barthelemy, MD (Department of Anesthesiology, Intensive care and Emergency Medicine, General and teaching Hospital of Nice); Michel Carles MD, PhD (Department of Anesthesiology, Intensive care and Emergency Medicine , General and teaching Hospital of Nice)  **Coordination of the study**: CT; Laurie Tran MD (Department of Anesthesiology, Intensive care and Emergency Medicine General and teaching Hospital of Nice)  **Surgery**: CT  **Development of study analysis**: CT; LT  **Writing manuscript**: CT; LT  **Revising manuscript**: LT; MB ; Pascal Boileau MD (iULS - University Institute of Locomotion & Sports, Department of Orthopedics & Sports , General and teaching Hospital of Nice); Marc Raucoules (Department of Anesthesiology, Intensive care and Emergency Medicine General and teaching Hospital of Nice); MC; CT. |
|  | 5b | AERCOT association, 151 Route ST ANTOINE DE GINESTIERE  06200 NICE, France |
|  | 5c | AERCOT provides to investigators the necessary information to conduct the clinical trial. AERCOT obtained all the necessary ethic reviews and approvals. AERCOT is ensuring compliance with labelling, reporting and record-keeping requirements and also ensuring that the clinical study is conducted in accordance with Good Clinical Practice. Data management is ensured by Laurie Tran, MD, |
|  | 5d | Not applicable |
| Introduction |  |  |
| Background and rationale | 6a | The number of patients operated for total knee arthroplasty (TKA) is growing worldwide. Outpatient surgery is defined by a length of stay (LOS) in the hospital inferior to 12 hours. This can be limited for TKA with the efficient management of pain and perioperative complications, such as blood loss, enabling a safe hospital discharge. Outpatient TKA with a suitable protocol, including multimodal measures, could improve the success rate of this procedure. Among the main measures, single shot sciatic nerve block with the association of continuous femoral nerve block for pain control need to be evaluated in outpatient TKA. Furthermore, to promote the safety of the postoperative period and to accelerate rehabilitation, patients who undergo ambulatory TKA could be discharged to a rehabilitation center the day of surgery to screen adverse events and to optimize the rehabilitation process. This study is designed to assess the benefits of sciatic nerve block in postoperative pain relief for outpatient TKA |
|  | 6b | Success rate of outpatient TKA (until the fifth postoperative day) |
| Objectives | 7 | The objective of this study is to assess a protocol for outpatient TKA including postoperative analgesia optimized to allow same-day discharge to a rehabilitation center |
| Trial design | 8 | Single-center randomized prospective controlled study, allocation ratio is 1 and framework of the study is superiority |
| Methods: Participants, interventions, and outcomes | | |
| Study setting | 9 | Academic hospital of Nice, France |
| Eligibility criteria | 10 | To be eligible, participants must meet the following inclusion criteria:   - Scheduled primary unilateral total knee arthroplasty - Acceptance of outpatient procedure - Patient age above 18 years and under 75 years - Normal weight or moderate obesity - ASA 1-2 - Patients without cognitive disorders having a good level of understanding of outpatient procedure (pain control) - No major thromboembolic episode in medical history - No contraindications for anesthesia or analgesia - Preoperative hemoglobin> 13 g/dl   Exclusion criteria will include:   - Bilateral total knee arthroplasty - Revision total knee arthroplasty - Unicompartimental knee arthroplasty - Severe (BMI > 35) and morbid (BMI > 40) obesity - ASA 3-4 - Obstructive sleep apnea syndrome - Hypersensitivity or allergy to anesthesia drugs - Refusal of the outpatient procedure by the patient - Emergency surgery - Anticoagulant therapy |
| Interventions | 11a | For TKA, all patients usually receive continuous femoral nerve block with 2 mg/ml Ropivacaine. Two groups are defined randomly. Patients who receive sciatic nerve block with 20 ml of Ropivacaine 2 mg/ml are allocated to the "sciatic-block group "(BS+). Patients who will not receive sciatic nerve block are allocated in a second group (BS-). |
|  | 11b | No criteria for discontinuing or modifying allocated interventions for a given trial participant |
|  | 11c | No Strategies to improve adherence to intervention protocols |
|  | 11d | No relevant concomitant care and interventions that are permitted or prohibited during the trial |
| Outcomes | 12 | Primary outcome is the success rate of outpatient TKA. The success rate is defined by patients discharged from the hospital to a rehabilitation center the day of surgery with no re-hospitalization due to insufficient pain control until the fifth postoperative day. Secondary outcomes include the incidence of adverse events before the fifth postoperative day (including hematoma, anemia, thromboembolism event, surgical site infection) and measurement of quality of recovery using the QoR-40 questionnaire |
| Participant timeline | 13 | Enrolment, during anesthesia visit, allocation to group the day at surgery, intervention in operating room the day of surgery. Assessments: day of surgery, day 1 to day 5, and visits for participants at 4 weeks, 3 months and at one-year. |
| Sample size | 14 | Calculation of sample size was based on previous studies.  We assumed that the failure of outpatient TKA procedure, defined by no discharge the day of surgery or re-hospitalization during the postoperative period due to pain could reach the peak value of 55 %.  The association of sciatic nerve block and continuous femoral nerve block could decrease this failure rate up to 15%. Group sample sizes of 19 for each group will achieve 80% power to detect this difference rate of outpatient procedure failure with an α risk of 5%. To anticipate any protocol dropout, we will include 40 patients (20 in each group) |
| Recruitment | 15 | Trial participants are recruited among patients of Professor Christophe Trojani, iULS - University Institute of Locomotion & Sports, Department of Orthopedics & Sports, General and teaching Hospital of Nice. |
| **Methods: Assignment of interventions (for controlled trials)** | | |
| Allocation: |  |  |
| Sequence generation | 16a | Computer-generated random numbers, no factors for stratification. |
| Allocation concealment mechanism | 16b | Mechanism of implementing the allocation sequence is sequentially numbered |
| Implementation | 16c | The allocation sequence is generated at the time of computer -generated random number. Trial participants are enrolled during anesthesia visit. Anaesthetist will assign participants to interventions the day of the surgery. |
| Blinding (masking) | 17a | Only trial participants will be blinded after assignment to interventions |
|  | 17b | No procedure for revealing a participant’s allocated intervention during the trial is planned. |
| **Methods: Data collection, management, and analysis** | | |
| Data collection methods | 18a | Specific case report form is used to collect data from trial. Data are reported on excel database and statistical analysis are performed using R studio ® software. |
|  | 18b | No plans to promote participant retention and complete follow-up, including list of any outcome data to be collected for participants who discontinue or deviate from intervention protocols |
| Data management | 19 | Data are anonymized using a specific procedure according to the protocol. The database is stored on a securized hospital computer server |
| Statistical methods | 20a | Categorical variables are expressed as percentages with their 95% confidence intervals  Continuous variables are expressed as mean ± standard deviation (SD), median and interquartile range (25th and 75th centiles)  Chi-square test or Fisher’s exact test (if n<10) were used to compare frequencies. T-test and Wilcoxon test were used to compare quantitative data according to the normality of the distribution.  The significant degree for p was set at 0.05. |
|  | 20b | No method for any additional analyses |
|  | 20c | Analysis are performed in a per-protocol way. No methods to handle missing data (missing at random data) |
| **Methods: Monitoring** | | |
| Data monitoring | 21a | Data monitoring is performed by specific team of University Institute of Locomotion & Sports, Department of Orthopedics & Sports, in General and teaching Hospital of Nice. |
|  | 21b | No interim analyses are planned. Decision to terminate the trial is planned when all included patients ends their follow-up |
| Harms | 22 | All adverse events and other unintended effects of trial interventions or trial conduct are reported and collected and transmitted to research committees |
| Auditing | 23 | No frequency and procedures for auditing trial conduct but unannounced check by independent supervisor. |
| Ethics and dissemination | | |
| Research ethics approval | 24 | Approval by research ethics may be request to principal investigators |
| Protocol amendments | 25 | Protocol amendments should be accepted by research ethics committee if needed |
| Consent or assent | 26a | Informed consent is obtained by the anaesthesiologist during anesthesia visit |
|  | 26b | Not applicable |
| Confidentiality | 27 | Personal information about enrolled patients will be anonymized and will be stored by the PI at least legal time of fifteen years |
| Declaration of interests | 28 | No competing interests for principal investigators for the overall trial and each study site |
| Access to data | 29 | Only data manager and PI have access to the final trial dataset. Database may be published if needed |
| Ancillary and post-trial care | 30 | Provisions, if any, for ancillary and post-trial care, and for compensation to those who suffer harm from trial participation according to the French law |
| Dissemination policy | 31a | Trial results will be communicated to healthcare professionals, via medical publication and through medical congress |
|  | 31b | No professional writers |
|  | 31c | Public access to the full protocol, participant-level dataset, and statistical code may be available on demand |
| Appendices |  |  |
| Informed consent materials | 32 | Model consent form in French |
| Biological specimens | 33 | Not applicable |

*Inform consent materials*

**Note d’information**

« Faisabilité de la prothèse totale du genou en Ambulatoire : apport du bloc sciatique »

Nom du Promoteur : Association AERCOT - Institut Universitaire Locomoteur et du Sport Service de Chirurgie Orthopédique et Chirurgie du Sport - Hôpital Pasteur 2 - 30 Ave Voie Romaine - CS 51069 - 06001 Nice Cedex 1 - CHU de Nice - Tel: 04.92.03.69.04

Investigateur principal à joinder en cas d’urgence 7/7 j et 24/24 heures: - Pr C Trojani – Responsable du Service de Chirurgie Orthopédique du genou au sein de l’Institut Universitaire Locomoteur et du Sport - Hôpital Pasteur 2 - 30 Ave Voie Romaine - CS 51069 - 06001 Nice Cedex 1 - CHU de Nice - Tel : 04.92.03.69.04 .

Numéro EudraCT : 2016-A00069-42.

Numéro National ANSM : 160095A-31.

**Nous vous demandons de lire attentivement ce document avant de donner votre accord de participation**

Madame, Monsieur,

Vous allez bénéficier au sein du service de chirurgie orthopédique de l’IULS du CHU de Nice, d’une prothèse totale de genou.

Ce type d’intervention est classiquement réalisé dans notre service sous rachianesthésie comme cela vous a été proposé par votre médecin anesthésiste, c'est-à-dire que seul le bas de votre corps (membres inférieurs) sera anesthésié.

Ce type d’intervention peut être source de douleurs post-opératoires importantes et nous faisons de la prise en charge de la douleur une de nos priorités. C’est pourquoi vous allez bénéficier en plus de la rachianesthésie, d’une anesthésie loco-régionale par injection d’anesthésique local à proximité des nerfs responsables de la douleur du genou sous contrôle échographique afin d’optimiser la prise en charge analgésique post-opératoire et d’en prolonger la durée sans avoir besoin d’antalgiques de pallier III (exemple la morphine) et de vous exposer à ses effets indésirables comme cela vous a été proposé par votre anesthésiste lors de la consultation.

Le contrôle optimal de la douleur permettra de réaliser cette chirurgie en ambulatoire ce qui signifie que vous entrerez le matin de la chirurgie et en sortirez en fin d’après-midi vers le centre de rééducation d’ Atlantis. Ceci vous sera bénéfique car il a été démontré qu’une durée d’hospitalisation la plus courte possible était avantageuse pour le patient : réduction des complications liés à l’alitement prolongé (réduction du risque de survenue de caillots, et de la perte musculaire), une réduction du temps nécessaire à la rééducation limitant ainsi la perte d’autonomie, une convalescence accélérée et un meilleur confort « psychologique » dans un environnement non hospitalier (centre de rééducation, domicile). Vous ne pouvez être inclus dans cette étude que parce que vous remplissez des critères précis permettant de vous proposer une prothèse totale du genou en ambulatoire. Cette stratégie ne peut être pas être proposée à tous les patients. En particulier, les patients ayant des antécédents cardio-vasculaires (infarctus, syndrome d’apnée du sommeil, phlébite, embolie pulmonaire), les patients déments, allergiques, prenant des anticoagulants, les patients ayant eu un cancer ne pourront pas participer à cette étude. Vous faites partie d’une population sélectionnée qui peut entrer dans notre protocole d’étude.

Nous vous proposons de participer à un protocole de recherche biomédicale qui a pour but l’amélioration de la prise en charge de la douleur après une prothèse totale du genou, grâce à la réalisation d’une anesthésie locale au niveau des nerfs responsable de la douleur du genou et d’une rachi-anesthésie, afin de réaliser cette chirurgie en ambulatoire.

Pour cela, tous les patients bénéficieront d’une rachianestésie (avec injection de Bupivacaïne) et d’une anesthésie locale sous échographie au niveau du nerf fémoral qui est un nerf responsable de la douleur du dessus du genou où il sera déposé un petit cathéter pour permettre une injection continue de Ropivacaïne®.. Ces médicaments (Ropivacaïne®, Bupivacaïne) sont régulièrement utilisés en médecine et depuis de nombreuses années. Aucun n’est un nouveau médicament et ces différents médicaments sont quotidiennement utilisés en anesthésie loco-régionale en France et dans le Monde.

En plus de ces deux méthodes d’anesthésie très fiables, une anesthésie locale sous échographie au niveau du nerf sciatique pourra être ajouté, c’est le nerf responsable de la douleur du derrière du genou. C’est la réalisation de cette anesthésie locale qui sera tirée au sort à l’arrivée au bloc opératoire. Le protocole prévoit donc deux groupes de patients: un groupe bénéficiera d’une d’une rachi-anesthésie et d’une anesthésie au niveau du nerf fémoral ; l’autre bénéficiera en plus de la rachi-anesthésie et de l’anesthésie du nerf fémoral, d’une anesthésie locale au niveau du nerf sciatique. Tous les patients auront donc une prise en charge de la douleur optimale et c’est l’intérêt de réaliser une anesthésie locale au niveau du nerf sciatique qui est à l’étude dans ce protocole.

La durée de l’étude prévue pour chaque patient sera de 3 mois comme cela est expliqué dans le tableau synoptique de l’étude (ci-dessous).

A 1 mois et 3 mois post-opératoire il est prévu une consultation chirurgicale qui a pour but d’étudier : la mobilité du genou, le niveau de douleur, l’état de la cicatrisation, le traitement anticoagulant, une surveillance biologique et un contrôle radiologique de la prothèse totale de genou.

A 1 mois post-opératoire il est prévu une consultation d’anesthésie qui a pour but : l’évaluation de l’impression globale de votre prise en charge, votre satisfaction et votre confort en post-opératoire immédiat et à distance.

Ces consultations ont pour but la recherche et l’élimination des effets indésirables ou de complications liés à la pratique de l’anesthésie et de la chirurgie pour prothèse totale de genou.

Les risques présentés par cette étude sont identiques à ceux de la prise en charge actuelle validée pour la pratique de l’anesthésie loco-régionale et pour ce type de chirurgie. En cas de problème pendant et après l’intervention, la prise en charge médicale est immédiatement adaptée à la situation d’urgence qui se présente et vous êtes alors réorientés vers le service d’hospitalisation traditionnelle pour assurer la suite de votre prise en charge.

Les bénéfices personnels attendus sont ceux déjà précités sur les avantages d’une réhabilitation précoce post-opératoire c’est à dire de supprimer tous les événements indésirables liés à une hospitalisation traditionnelle. Il s’agit de mettre le patient au centre de sa prise en charge comme acteur de ses soins autour duquel s’articule une équipe pluridisciplinaire dont l’objectif est d’accélérer sa convalescence et réduire significativement sa durée moyenne d’hospitalisation. Au niveau collectif, cette étude permettra d’améliorer la prise en charge analgésique post-opératoire et de développer la réalisation des prothèses totales du genou en ambulatoire dans le cadre d’une étude strictement contrôlée par la loi, pour un groupe de patient sélectionné.

Il n’y a aucune modification dans votre prise charge médicale habituelle du fait de votre participation à cette étude. Un test de grossesse urinaire sera effectué pour les femmes en état de procréer. Le résultat du test vous sera communiqué. Dans le cas d’un résultat positif, vous ne pourrez être incluse dans l’étude.

La participation à cette recherche est volontaire, vous pouvez vous en retirer à tout moment sans que cela ne modifie votre prise en charge et les soins qui vous sont prodigués. Par ailleurs, vous pouvez à tout moment avoir la possibilité d’accès à vos données et en demander le transfert au médecin de votre choix. Toutes les données et informations vous concernant resteront strictement confidentielles. Les données vous concernant ne seront accessibles qu’aux personnes participant à cette recherche et aux personnes chargées par le promoteur de contrôler la qualité de l’étude ; le cas échéant elles pourront également être transmises aux autorités sanitaires habilitées. Dans tous les cas, elles seront exploitées dans les conditions garantissant leur confidentialité et seront protégées par le secret professionnel ainsi que par la loi informatique et libertés n°2004-801 du 6/08/2004 et le décret d’application numéro 2005-1309 paru le 20/10/2005.

Vous aurez le droit de vous opposer au traitement automatisé des données vous concernant.

Les données de l’étude pourront être publiées dans des journaux médicaux et scientifiques sans que votre identité soit révélée. Le promoteur est responsable du traitement des données de l’étude.

La durée de l’étude n’excède pas 1 an. Vous pouvez vous opposer à tout moment à l’utilisation des données vous concernant et vous retirer à tout moment de l’étude, sans avoir à vous justifier et sans que cela modifie en quoi que ce soit votre prise en charge médicale.

Il n’est pas prévu de période d’exclusion à la suite de votre participation à cette étude.

Les résultats globaux de la recherche à laquelle vous participez, pourront vous être communiqués par courrier à votre adresse personnelle.

Il est prévu une interdiction de participer simultanément à une autre recherche pendant toute la période de cette étude.

Le promoteur a contracté une assurance en responsabilité civile auprès de la Société Hospitalière d’Assurance Mutuelle (SHAM), contrat n° 141.402, pour la réalisation de cette étude.

Vous avez la possibilité de poser toutes les questions que vous souhaitez.

Cette recherche a reçu l’avis favorable du Comité de protection des personnes CPP Sud Méditerranée V le 07 juin 2016 et une autorisation de l’Agence Nationale de Sécurité du Médicament et des produits de santé (ANSM) le 15 avril 2016, numéro national de référence : 160095A-31.

Paraphe de l’investigateur Paraphe de la personne

**Recueil du consentement**

**«**Faisabilité de la prothèse totale du genou en Ambulatoire : apport du bloc sciatique »

*Les détails concernant cette étude sont fournis dans la lettre d’information spécifique qui vous a été remis. Lisez attentivement cette notice et posez toutes les questions qui vous sembleront utiles. Si vous acceptez de participer à cette étude, veuillez compléter le formulaire ci-dessous.*

Je soussigné (e),

Mme, Mlle, M. (rayer les mentions inutiles) ……………………………………… (nom complet en lettres capitales) déclare avoir compris le but et les modalités de cette étude, qui m’ont été pleinement expliqués par le Docteur …………..……………………………………

J’ai reçu le formulaire d’information spécifique que j’ai eu la possibilité d’étudier avec attention. On a répondu à toutes mes questions et j’en suis satisfait(e). J’ai disposé d’un délai de réflexion suffisant avant de prendre ma décision.

J’accepte de mon plein gré de participer à cette recherche dans les conditions précisées dans le formulaire d’information ci-joint. Je sais que je pourrai, à tout moment, retirer mon consentement sans que cela n’affecte mon traitement ultérieur. J’en informerai alors le Docteur …………………………………… Le fait de ne plus participer à cette recherche ne portera pas atteinte à mes relations avec mon médecin et ne remettra pas en cause la qualité des soins ultérieurs.

J’ai été informé(e) que conformément à la réglementation sur les études cliniques, le Comité de Protection des Personnes « Sud Méditerranée V » a rendu un avis favorable et que l’Agence Nationale de Sécurité du Médicament et des produits de santé (ANSM) a délivré une autorisation pour la réalisation de cette étude.

Toutes les données me concernant, y compris mon dossier médical, resteront confidentielles. Je n’autorise leur consultation que par les personnes qui collaborent à la recherche, aux personnes chargées par le promoteur de contrôler la qualité de l’étude ainsi que par un représentant des autorités de santé.

J’accepte que les données nécessaires à la recherche soient recueillies durant ma participation à l’étude, et fassent l’objet d’un traitement informatisé autorisé par la Commission Nationale Informatique et Liberté. J’ai bien été informé de la finalité du traitement (on m’a expliqué à quoi serviraient ces données) ainsi que des destinataires de ces données.

J’ai bien noté qu’en application de la loi « Informatique et Libertés » du 6 janvier 1978 modifiée et la loi du 04 mars 2002 relative aux droits du malade, je dispose d’un droit d’accès aux données me concernant ainsi qu’un droit de rectification. Je peux exercer ces droits à tout moment auprès du Dr………………Service………………Hôpital ………………………..Tél…………..…………

Je donne mon consentement pour participer à cette recherche en toute connaissance de cause et en toute liberté.

Je pourrai à tout moment demander toute information complémentaire au Dr…….…………..…..

N° de téléphone ………..…………

Mon consentement ne décharge en rien l’investigateur et le promoteur de l’ensemble de leurs responsabilités et je conserve tous mes droits garantis par la loi.

A l’issue de la recherche, je pourrai être informé(e) des résultats globaux de cette recherche par courrier.

| A REMPLIR PAR LE PATIENT |
| --- |
| Date : ………  Signature du patient |

| A REMPLIR PAR L’INVESTIGATEUR | |
| --- | --- |
| Je soussigné Docteur …………….…………………. (nom en lettres capitales) confirme avoir pleinement expliqué au patient le but et les modalités de cette étude ainsi que ses risques potentiels. Je m’engage à faire respecter les termes de ce formulaire de consentement, conciliant le respect des droits et des libertés individuelles et les exigences d’un travail scientifique.  N° de téléphone de l’investigateur : …………………………………….. | |
| Signature de l’investigateur : | Date : …………………………….. |

Fait en deux exemplaires dont un sera conservé par l’investigateur et un autre remis au patient.
